# Fiber effects in nutrition and gut health in pigs

**DOI:** 10.1186/2049-1891-5-15

**Published:** 2014-03-01

**Authors:** Jan Erik Lindberg

**Affiliations:** 1 Department of Animal Nutrition and Management, Swedish University of Agricultural Sciences, P.O. Box 7024, Uppsala SE-75007, Sweden

**Keywords:** Breed, Gut health, Fiber, Pigs, Prebiotics, Utilization

## Abstract

Dietary fiber is associated with impaired nutrient utilization and reduced net energy values. However, fiber has to be included in the diet to maintain normal physiological functions in the digestive tract. Moreover, the negative impact of dietary fiber will be determined by the fiber properties and may differ considerably between fiber sources. Various techniques can be applied to enhance nutritional value and utilization of available feed resources. In addition, the extent of fiber utilization is affected by the age of the pig and the pig breed. The use of potential prebiotic effects of dietary fiber is an attractive way to stimulate gut health and thereby minimize the use of anti-microbial growth promoters. Inclusion of soluble non-starch polysaccharides (NSP) in the diet can stimulate the growth of commensal gut microbes. Inclusion of NSP from chicory results in changes in gut micro-environment and gut morphology of pigs, while growth performance remains unaffected and digestibility was only marginally reduced. The fermentation products and pH in digesta responded to diet type and were correlated with shifts in the microbiota. Interestingly, fiber intake will have an impact on the expression of intestinal epithelial heat-shock proteins in the pig. Heat-shock proteins have an important physiological role in the gut and carry out crucial housekeeping functions in order to maintain the mucosal barrier integrity. Thus, there are increasing evidence showing that fiber can have prebiotic effects in pigs due to interactions with the gut micro-environment and the gut associated immune system.

## Introduction

On a worldwide basis corn and soybean meal are the main staples in the diet for pigs and poultry, providing most of the energy and nutrients needed. It is argued that although other cereals, such as wheat, and by-products, such as rice bran and distiller’s grains, are used as alternative feedstuffs in part of the world the quantities available are not sufficient to replace corn and soybean meal in the global pig and poultry industry [1]. This may be correct and may apply to the industrialized livestock production, but may not be applicable to countries where a major part of the livestock production relies on smallholder farmers.

With more focus on small-scale family farming, in order to improve food security and minimize the negative impact on the environment and the climate, there are opportunities for more diversified composition of the diet with respect to feedstuffs included. Potential feed resources used for animals in many countries in Asia derive primarily from the vegetable foods and agro-industry co-products, such as cassava leaves, sweet potato vines, water spinach, rice bran, cassava residue, brewer’s grain and tofu residue. They represent under-utilized feedstuffs, most having high fiber content, which may impose limitations in their use in diets for mono-gastric animals, in particular young animals, due to their bulky nature and a limited capacity to ferment fiber [2]. Thus, in order to better utilize available fiber-rich feedstuffs in the diet, their chemical and physical characteristics has to be described and taken into account in feed formulation.

Dietary fiber has an important role in pig and poultry diets and a minimum level of dietary fiber has to be included to maintain normal physiological function in the digestive tract [3]. A major concern when including fiber in diets for mono-gastric animals is that high dietary fiber content is associated with decreased nutrient utilization and low net energy values [4]. However, the negative impact of dietary fiber on nutrient utilization and net energy value will be determined by the fiber properties and may differ considerably between fiber sources. Moreover, dietary fiber may have other positive effects such as to stimulate gut health, increase the satiety, affect behavior and overall improve animal well-being [2,3,5,6]. Despite the obvious need for dietary fiber in the diet it is not included in the nutrient requirement tables [7].

For a long time antibiotics have been used as growth promoters on a regularized basis to control the problems with commonly occurring enteric diseases, such as post-weaning diarrhea and swine dysentery [6]. However, this is not a sustainable production system and the regular use of antibiotics in animal feed will promote bacterial resistance that may result in less efficient antibiotic treatments for human and animal diseases [8,9]. In addition, misusing antibiotics as feed additives for animal production can result in high antibiotic residues in animal products. Since 2006 antimicrobial growth promoters (AMGP) have been banned within EU and the goal is minimal use of antibiotics in food production. A similar development can be expected to take place also in other parts of the world.

In order to minimize the use of AMGP in animal production it is important to find ways to obtain a good animal health with other means. One possible way is changing diet composition and to use various dietary interventions. A huge amount of research has been performed in the area that aims to change the gut microbial composition, such as supplementation of probiotics, prebiotics, organic acids and dietary fiber [6]. It is believed that these types of dietary interventions have the potential to improve gut health.

### Dietary fiber

The carbohydrate fraction can be divided according to glycosidic linkages into sugars, oligosaccharides and two broad classes of polysaccharides, starch and non-starch polysaccharides. Non-starch polysaccharides (NSP) together with lignin, has been defined as the dietary fibre (DF) fraction in feedstuffs and food, and can be used as a collective measure of their fibre content [10-12]. However, as non-digestible oligosaccharides and resistant starch have similar physiological effects in the body as NSP and lignin, although not being part of the cell wall structure, the definition should be extended to include these constituents [13].

The amount and composition of dietary fibre vary widely between and within feedstuff [12,14,15]. As a result, the dietary fibre content and its properties in a typical diet for any animal varies between production systems and countries due to availability of feed resources. A change in chemical composition of the plant material and their co-products not only depends on the botanical origin of the plants and the type of processing applied, but also on the tissue type and the maturity of the plant at harvesting time. The proportion of middle lamella, and primary and secondary cell wall in the plant material, together with the properties of the amorphous matrix connecting the cell walls, will determine both utilization and properties. The amorphous matrix usually shows considerable variation from tissue to tissue within plant and between plants. In monocotyledonous plants, such as cereals, the main cell wall NSP are arabinoxylans, cellulose and β-glucan [12]. In contrast, the amorphous matrix in dicotyledonous plants can differ markedly from that in monocotyledonous plants due to different tissue types, exemplified by the huge difference in content and properties of pectic substances [16,17].

**Figure 1:**
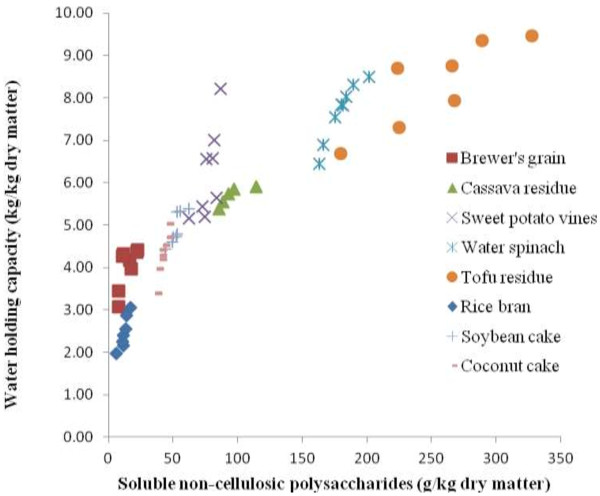
**Correlation between water holding capacity (WHC, kg/kg DM) and soluble non-starch polysaccharides (S-NCP, g/kg DM) in selected fibre-rich plant sources and agro-industry co-products (WHC = 3.50 + 0.0214 S-NCP, R**^**2**^ **= 0.82, *P*** **< 0.001) (Ngoc et al.**[15]**).**

The physicochemical properties of dietary fibre are dependent on the polysaccharides that make up the cell wall and their intermolecular association which determine their solubility [17]. The major physicochemical properties that have been considered in animal nutrition are cation exchange capacity, hydration properties, viscosity and organic compound adsorptive properties. Characteristic for insoluble dietary fiber is that they increase rate of passage and faecal bulk whereas soluble dietary fiber increases the viscosity and hydration properties [18]. Hydration properties of the feed is very important for effective digestion to occur in the animal and can be characterized by the swelling capacity, solubility, water holding capacity (WHC) and water binding capacity (WBC). Recent studies showed a strong correlation between content of soluble non-cellulosic polysaccharides (S-NCP) and WHC in plant material and agro-industry co-products collected in Vietnam [15]. This could be due to the occurrence of more gaps within the cell matrix that can retain excess water in feed ingredients which are high in S-NCP. Similarly, it has been shown that the S-NCP fraction in co-products from vegetable food and agro-industries was linearly related to selected hydration properties, such as swelling and WBC [14] (Figure 1).

### Nutritional effects

### Improving feed value

Various techniques, such as pelleting, reduction of particle size and supplementation with exogenous enzymes can be applied to enhance nutritional value and utilization of available feed resources [19-21]. This may be most relevant for locally available feed resources to overcome the shortcomings of poor nutritive value [6,22] due to high fibre content and of other components in the feed affecting the physicochemical properties.

The positive effects of enzyme addition is due to disruption of intact cell walls and release of entrapped nutrients or due to the changes in the physical properties of non-starch polysaccharides, such as viscosity and WHC, and/or changes in the composition and content of bacteria in the small and large intestine [23-25].

Reducing particle size has been reported to improve the feed efficiency and nutrient digestion of weaned pigs [19,21]. There appears to be a particle size by age of pig interaction in digestibility, resulting in a larger response in young as compared to older pigs [19]. This can be explained by the development of the digestive and absorptive capacity in the small intestine and increased colonization of carbohydrate-degrading microbiota in the large intestine with increasing age. There are also indications of a particle size by enzyme interaction for the total tract apparent digestibility of dietary components in pigs [21].

Reduction in particle size (1 versus 3 mm) increased the total tract apparent digestibility of dietary components and the average daily gain (ADG) of Landrace x Yorkshire pigs in the post-weaning period, but not in the growing period [26]. Moreover, addition of a multi-enzyme mixture (mixture of α-amylase, β-glucanase, cellulase and protease) improved the total tract apparent digestibility of dietary components and growth performance in the post-weaning period [26]. However, there was an interaction between particle size and multi-enzyme supplementation on the total tract apparent digestibility of crude protein (CP) and neutral detergent fibre (NDF) in the post-weaning period, such that multi-enzyme supplementation increased the total tract apparent digestibility of CP and NDF in the larger particle size diet (80% vs. 75%, and 58% vs. 51%, respectively), while there was no changes in a small particle size diet (80% vs. 78%, and 59% vs. 55%, respectively). However, in the growing period multi-enzyme supplementation had no positive effect on the performance and the total tract apparent digestibility of dietary components, with the exception of CP and NDF (Table 1).

**Table 1:** **Impact of fiber source, particle size and enzyme treatment on dry matter intake (DMI), average daily gain (ADG) and feed conversion ratio (FCR) in post-weaned and growing pigs**^**1**^

**Items**	**Post-weaned pigs**			**Growing pigs**		
	**DMI**	**ADG**	**FCR**	**DMI**	**ADG**	**FCR**
Fiber source						
Cassava root meal	559	422^a^	1.33^a^	1289	597^a^	2.16^a^
Sweet potato vines	550	385^b^	1.44^b^	1271	540^b^	2.36^b^
Particle size						
Small (1 mm)	556	416^a^	1.34	1273	572	2.24
Large (3 mm)	553	391^b^	1.42	1286	565	2.29
Enzyme addition^#^						
-	554	387^b^	1.44^b^	1265	557	2.28
+	555	420^a^	1.33^a^	1295	580	2.24
SEM	28	12	0.07	41	17	0.08

^1^Data from Ngoc et al. [26]. Different letters within column indicates significant differences between treatments. SEM = standard error of the mean.

^#^Multi-enzyme mixture (α-amylase, β-glucanase, cellulase and protease).

### Fiber utilization

#### ***Impact of age***

Adult pigs have a more developed and larger GI tract, a lower feed intake per kg body weight, a slower digesta transit time and a higher cellulolytic activity than young pigs. This resulted in greater capacity of sows to digest fibrous components compared to young pigs [27] and it was shown that sows digest a larger part of the NSP in the small intestine than growing pigs [28]. They also showed that sows have a higher capacity to digest insoluble NSP, whereas the difference in digestibility of soluble NSP between growing pigs and sows were only marginal. It was suggested that because of the increased capacity to digest fibrous feedstuff by increased age and body weight, at least two different energy values, one for growing-finishing pigs and one for sows should be used for most feed ingredients in pig diets [4]. This has been implemented in the INRA net energy system for pigs (http://www.evapig.com).

In weaned pigs, age affected the total tract apparent digestibility of all dietary components except for NDF, with higher values at five than at three weeks post-weaning [29].

#### ***Impact of pig breed***

Digestive capacity and potential body protein deposition are two important traits associated with pig performance. However, in commercial pig breeding research has mainly focused on factors associated with body lipid and protein deposition [30,31], while less attention has been directed to breed differences in the digestive capacity [32]. Thus, body protein and lipid mass and their deposition rates are key variables used in growth models to predict compositional changes in carcass muscle and fat tissue mass over time [30,33]. This priority can be explained by the increasing demand of consumers for meat quality in general and for lean meat in particular. It is also reflected in an increasing use of exotic breeds such as the Landrace, Yorkshire, Duroc and Hampshire for cross-breeding of native pigs, mainly in urban and peri-urban areas, in several countries in Asia. Crossbred Large White × Yorkshire (LY) pigs show higher growth rate and better feed conversion than native Mong Cai (MC) pigs, when fed the same daily amount of DM and CP [34,35]. This is due to greater potential for lean tissue accretion in LY than in MC pigs, as reflected in higher nitrogen retention [34,35].

**Figure 2:**
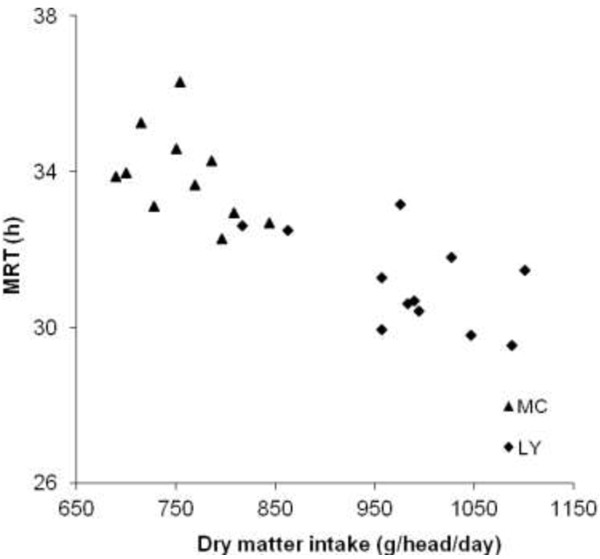
**Impact of dry matter intake (g/head/day) on mean retention time (MRT; h) of digesta in growing Mong Cai (MC) and Landrace x Yorkshire (LY) pigs (Ngoc et al.**[37]**).**

However, indigenous breeds, such as the MC breed, may have better characteristics with regards to reproduction and are adapted to the local climate. Therefore, in Vietnam pure-bred MC sows are still commonly mated with boars of an exotic breed, usually Yorkshire or Landrace, using artificial insemination, and the off-springs are fattened in small-scale semi-intensive or intensive systems. Moreover, it has been shown that indigenous pig breeds may have a higher capacity to digest fibre than exotic pig breeds genetically improved to support high growth performance [22,34-36]. The main explanation to the improved digestibility in indigenous pigs is a longer MRT of digesta [37]. This will contribute to a more efficient digestion due to longer contact between digesta, digestive enzymes and absorptive surfaces [32], and between digesta and the gut microbiota [38]. A longer MRT in the indigenous MC pig can be explained by a larger GIT (in terms of diameter), reflected in more gut content, as compared to LY pigs. In accordance, it was reported that differences in gut content could explain the longer MRT in Iberian pigs than in Landrace pigs [39]. Earlier studies on indigenous and exotic pig breeds support the contention of greater length of the GIT in the indigenous pigs [36,40] (Figure 2).

In addition, recent studies show that there are interactions between breed and diet on LAB count and in the concentration of propionic acid in the ileum. Increased fibre level in the diet in combination with high soluble fiber content had a greater impact on the LAB count and the concentration of propionic acid in the ileum of MC pigs than of LY pigs [41]. This suggests differences in the gut microbiota activity and/or composition between MC and LY pigs.

Interestingly, there was also a breed effect on the small intestinal morphology in the ileum, while there were no breed related differences in the duodenum and jejunum. Mong Cai pigs had shorter villi, smaller villus width and greater crypt density in the ileum than LY pigs [41]. These differences in gut morphology can be explained by differences in digesta transit time and gut microbial activity [42]. A major part of the digested nutrients are absorbed in the proximal small intestine. This together with a rapid digesta passage and low microbial activity, results in less exposure to digesta components compared with the situation in the more distal small intestine. The MC pigs had a longer total tract mean retention time of solids than the LY pigs [37] allowing for longer contact between digestion products and absorptive surfaces [32].

Based on existing data, it appears reasonable to assume that different breeds can have different digestive capacity and will as a result show different response to the same diets in terms of nutrient utilization and performance.

### Effects on gut health

### Gut microenvironment

The maintenance of gut health is complex and relies on a delicate balance between the diet, the commensal microflora and the mucosa, including the digestive epithelium and the mucus overlying the epithelium [43]. The diet has a great impact on gut health, and it can provide either beneficial or harmful input [2,6,44]. The diet should be composed to create a balance between the gut, the microbiota and the gut environment and prevent disturbances in the gut. Dietary fibre interacts both with the mucosa and the microbiota and consequently has an important role in the control of gut health [2,6,43].

**Figure 3:**
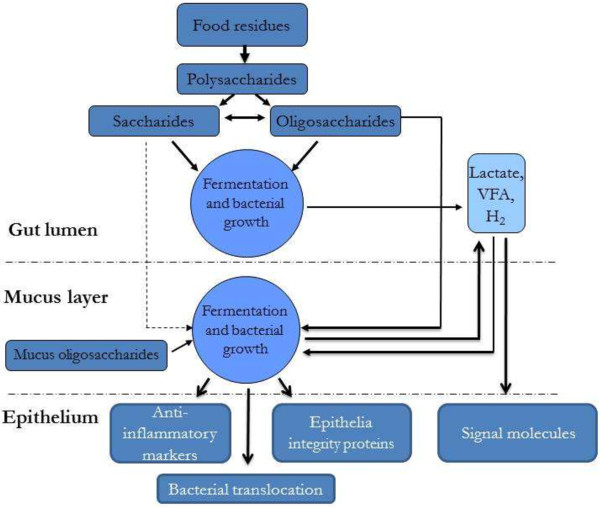
Interactions between dietary fiber, gut environment, gut microbiota and host response with implications on gut health.

The complex microbial community in the gastrointestinal (GI) tract consists of different groups of microbes including bacteria, archaea, ciliate and flagellate protozoa, anaerobic phycomycete fungi and bacteriophages. Bacteria are the most abundant and studied microbes in this community. They are provided with substrates from the diet as well as components deriving from the host such as mucopolysaccharides, mucins, epithelial cells and enzymes [45]. With the introduction of molecular techniques to indentify the microbiota it has become apparent that only a minority of the GI microbes have been isolated by culture based methods [46] and consequently the knowledge we have today most likely needs to be revised in the future (Figure 3).

### Prebiotic effects

The concept of prebiotics was initially defined as “non-digestible food ingredient that beneficially affects the host by selectively stimulating the growth and/or activity of one or a limited number of bacteria in the colon, and thus improves host health” [47]. However, the weakness with this definition is that almost every food oligosaccharide and polysaccharide (including dietary fibre) can be claimed to have prebiotic activity. Later, it was proposed that to be allowed to classify a compound as a prebiotic it should scientifically be demonstrated that it resists host digestion, absorption and adsorption processes, is fermented by the microbiota colonising the GI system and selectively stimulates the growth and/or the activity of one or a limited number of bacteria within the GI system [48].

Dietary fiber (DF) is a feed component that has major influence in this regard. Dietary fibre components are not digested by endogenous digestive enzymes, and consequently are the main substrates for bacterial fermentation in the distal part of the gut. The main products of fermentation are short chain organic acids (OA), predominantly lactate, acetate, propionate and butyrate. The OA have been suggested to develop the growth of the digestive tract, by stimulating epithelium cell proliferation [43]. In an acidic environment, OA can inhibit the growth of enteric bacterial pathogens, such as *Salmonella*, *E. coli* and *Clostridium* species [49-51]. Studies in pigs have shown that the various types of plant carbohydrates behave differently in the GIT depending on their structural characteristics. Inclusion of soluble NSP in the diet can stimulate the growth of commensal gut microbes, leading to increased production of OA, and a lower pH in the large intestine [2]. Insoluble NSP reduce the transit time and provide substrate that is slowly degradable by the microbiota in the distal large intestine [38], and modulate gut morphology by increasing villus length [52].

Feeding guar gum, a soluble and viscous NSP, increases the proliferation of enterotoxigenic *E. coli*[53], whereas feeding insoluble NSP reduces the occurrence of haemolytic *E. coli,* and reduces the severity of post weaning colibacillosis [54]. However, it was shown that soluble NSP per se is not detrimental to piglet health [55]. Instead it was stated that soluble NSP that does not increase the digesta viscosity may beneficially affect gut health by increasing the lactobacilli:coliform ratio and decrease the occurrence of weaning diarrhoea.

The impact of DF source on gut microbiota composition and gut micro-environment was nicely demonstrated in a recent study on chicory. The inclusion of chicory (*Cichorium intybus* L.) forage and root in a cereal-based diet (wheat and barley) results in changes in gut micro-environment and gut morphology of pigs [56], while growth performance was unaffected and digestibility was only marginally reduced by chicory inclusion [29,57]. Within diet type, these changes followed a similar pattern in the small and large intestine. However, the dietary responses were different with inclusion of chicory root compared with chicory forage. This could be related to the chemical composition of the dietary fiber fraction [57], where the root is characterized by high content of inulin-type fructan and oligofructose, while the forage is characterized by high content of pectin. The fermentation products and pH in digesta responded to diet type and were correlated with shifts in the microbiota, showing that chicory influences the intestinal micro-environment of pigs [56]. In ileum, inclusion of chicory roots (inulin-type fructan and oligofructose) was linked with lactic acid concentration in digesta and relative abundance of LAB. In colon, inclusion of chicory forage (pectin) was associated with relative abundance of butyrate-producing bacteria and colonic acetate concentration.

Interestingly, diet fiber intake will have an impact on the expression of intestinal epithelial heat-shock proteins (HSP) that have an important physiological role in the gut. The HSP carry out crucial housekeeping functions in order to maintain the mucosal barrier integrity. In a recent study [58] we found a positive correlation between ileal HSP27 expression and daily total uronic acid intake (r = 0.364, *P = 0.05*). This was reinforced when ileal HSP27 and daily soluble uronic acid intake was correlated (r = 0.390, *P = 0.048*). Furthermore, HSP27 expression in the ileal mucosa was correlated with mucosa-associated *Megasphaera elsdenii* (TRF275) (r = 0.553, *P = 0.021*), which was also positively correlated with daily total uronic acid intake (r = 0.523, *P = 0.011*). However, daily fructan intake was not correlated with HSP27 expression (*P > 0.0*5).

## Conclusions

The impact of dietary fiber on pig nutrition and health is determined by the fiber properties and may differ considerably between fiber sources. Moreover, the utilization of plant fiber can be improved by using various techniques and may be improved by breeding for enhanced fiber utilization. There are increasing evidence showing that fiber can have prebiotic effects in pigs due to interactions with the gut micro-environment and the gut associated immune system. This property can be exploited and used as a means to stimulate gut health and thereby minimize the use of anti-microbial growth promoters. In addition, fiber in the diet will increase the satiety, affect behavior and overall improve animal well-being.

## Competing interests

The author declares that there are no competing interests.
